# Thermal Resistance Variations of Fly Ash Geopolymers: Foaming Responses

**DOI:** 10.1038/srep45355

**Published:** 2017-03-27

**Authors:** Heah Cheng-Yong, Liew Yun-Ming, Mohd Mustafa Al Bakri Abdullah, Kamarudin Hussin

**Affiliations:** 1 Center of Excellence Geopolymer and Green Technology (CEGeoGTech), School of Materials Engineering, Universiti Malaysia Perlis (UniMAP), 01000, P.O. Box 77, D/A Pejabat Pos Besar, Kangar, Perlis, Malaysia; 2 Faculty of Engineering Technology, Universiti Malaysia Perlis (UniMAP), P.O. Box 77, D/A Pejabat Pos Besar, Kangar, Perlis 01000, Malaysia

## Abstract

This paper presents a comparative study of the characteristic of unfoamed and foamed geopolymers after exposure to elevated temperatures (200–800 °C). Unfoamed geopolymers were produced with Class F fly ash and sodium hydroxide and liquid sodium silicate. Porous geopolymers were prepared by foaming with hydrogen peroxide. Unfoamed geopolymers possessed excellent strength of 44.2 MPa and degraded 34% to 15 MPa in foamed geopolymers. The strength of unfoamed geopolymers decreased to 5 MPa with increasing temperature up to 800 °C. Foamed geopolymers behaved differently whereby they deteriorated to 3 MPa at 400 °C and increased up to 11 MPa at 800 °C. Even so, the geopolymers could withstand high temperature without any disintegration and spalling up to 800 °C. The formation of crystalline phases at higher temperature was observed deteriorating the strength of unfoamed geopolymers but enhance the strength of foamed geopolymers. In comparison, foamed geopolymer had better thermal resistance than unfoamed geopolymers as pores provide rooms to counteract the internal damage.

From the past decades, the concern on the thermal performance and fire resistance of building materials has become one of the research interests. The thermal stability is important to ensure that they are safe for usage at certain temperature range. In the case of ordinary Portland cement (OPC), it degrades in strength irreversibly starting at 200 °C. This is brought about by the loss of water and decomposition of the main binding phases, that are, calcium silicate hydrate (CSH), Ca(OH)_2_ and others hydrated products^1^. Nevertheless, according to Aydin *et al*.^2^, the decomposition of Ca(OH)_2_ did not lead to critical degradation of strength. Yet, the lime expansion during cooling is the main factor contributing to strength loss of OPC. Notwithstanding the high structural strength, OPC emits high amount of CO_2_ gas, has high energy consumption production process, poor chemical resistance and moderate fire resistance.

In succession, geopolymers have been proposed as alternative building materials to OPC due to their sustainability characteristics of being the low emission of greenhouse gases^3^ and low energy consumption manufacturing process^4^. Compared to OPC, geopolymers perform better properties such as excellent mechanical strength, lower shrinkage, fast setting and better resistance to chemical and fire^5^,^6^. In general, geopolymers are inorganic aluminosilicates materials formed through the dissolution and polycondensation of aluminosilicate sources in highly alkaline solution. The aluminosilicates sources for the synthesis of geopolymer can be of natural origins (e.g. kaolin^7^, metakaolin^8^,^9^ or volcanic ash^10^) or wastes (e.g. industrial ashes^11^,^12^ or slags^13^,^14^). The alkali medium is most commonly a mixture of silicate solution and an alkali hydroxide.

In recent years, research works dealing with the thermal behavioural of geopolymers exposed to high temperature or fire have been widely carried out^15^,^16^. Geopolymers decreased in strength when exposed to elevated temperature as OPC does. Even so, they retained much high bond strength in the tested temperature range^17^. Bernal *et al*.^18^ studied the high-temperature performance of slag/metakaolin geopolymer blends. Higher strength retention is associated with lower weight loss of geopolymers. At high temperature (600 °C), geopolymer mortars with high slag content exhibited greater strength loss, which is mainly due to the dehydration of CSH phases. Nonetheless, all blended geopolymer mortars retained strength between 23 and 25 MPa up to 600 °C exposure. Geopolymer concretes experienced a lesser reduction in weight but greater strength loss with increasing exposing temperatures than geopolymer mortar. It is because there is lower binder content in concretes to counteract the shrinkage of paste and due to the difference in the thermal expansion between coarse aggregates and binder that contributed to significant microcracking. They claimed that geopolymers have the better thermal endurance to ordinary concrete or even some high performance concretes.

Moreover, based on the work by Fernandez-Jimenez *et al*.^19^, fly ash geopolymers retained strength up to 400 °C and further increased in strength at temperature beyond 400 °C. Crystallization of thermally stable materials such as sodalite and nepheline were observed in alkali-activated fly ash. Upon exposed to elevated temperature, crystalline phases of nepheline were commonly detected from XRD diffractograms of geopolymer samples^20^,^21^. The presence of thermally stable crystalline phases is crucial for the thermal stability of geopolymer structure^19^. Besides, solidification of melted phases also contributed to the strength improvement. Comparatively, OPC retained compressive strength up to 600 °C and reduced sharply beyond 600 °C due to loss of moisture and decomposition of Ca(OH)_2_ content. According to Rickard *et al*.^22^, the compressive strength of geopolymer was greatly dependent on the conversion of amorphous aluminosilicates into geopolymer structure. The durability towards elevated temperature and firing was affected by the Si/Al ratio and iron content in the fly ash.

Based on Duan *et al*.^23^, metakaolin-fly ash based geopolymers exhibited strength of 46 MPa at 1000 °C. With the addition of electrical porcelain as aggregates, the high-temperature performance enhanced^6^. In addition, Kamseu *et al*.^24^ studied the thermal stability up to 1200 °C of potassium-based metakaolin geopolymers in term of shrinkage and microstructural changes. With the addition of α-quartz sand or alumina powder, the maximum densification temperature increased. Comparison between metakaolin and fly ash geopolymers has shown that fly ash geopolymers are more resistant towards elevated temperature^25^,^26^. The fibres such ash wollastonite and basalt fibres could also be added in order to enhance the thermal properties of geopolymers^27^.

Besides, porous materials could also provide some sort of thermal barrier^28^. The development of lightweight porous materials has become one of the important research interests. Lightweight building materials offer benefits such as rapid construction, better thermal efficiency and fire resistance. Lightweight porous geopolymer materials (or geopolymer foams) can be obtained through foaming process by introducing small pores (closed cells) or interconnected voids (open cells) inside the material. The foam could be introduced through air bubbles^20^ or endogeneous gas generation (e.g. aluminium powder^29^, hydrogen peroxide^30^,^31^ or sodium hypochlorite^32^,^33^). In this study, hydrogen peroxide was used as the gas-forming agent. Under alkaline environment, hydrogen peroxide decomposes into water and oxygen as shown in Equations (1)^5^,^6^.


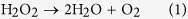


In term of the thermal properties of foamed geopolymer, Badanoiu *et al*.^34^ observed the volume increment when exposed the foamed geopolymers based on glass cullet and red mud at temperature between 600 °C and 800 °C. The foamed geopolymer had an apparent density lower than 866 kg/m^3^ with strength of > 2 MPa. Higher strength (2–30 MPa) foamed fly ash geopolymers with density lower than 1000 kg/m^3^ was produced by Zhang *et al*.^20^ with 30% of slag substitution. The fly ash geopolymer foam showed good strength retention up to 400 °C and further increased in strength when heated to 800 °C. According to Skvara *et al*.^29^, the geopolymer foam did not collapse or disintegrate below 1000 °C. High thermal resistance at elevated temperature is associated with the high shrinkage and sintering effect^35^. To our knowledge, there are very less literature on the thermal performance and fire resistance of foamed geopolymer materials. The porous geopolymer foam is usually assumed to have the same thermal behaviour as the dense geopolymers when exposed to elevated temperature and fire.

Thus, in this study, a comparative study is carried out to investigate the thermal behaviour of unfoamed and foamed geopolymers based on the fly ash from a local coal combustion power plant.

## Experimental

### Materials

Class F Fly ash was used as the aluminosilicate source. The fly ash was collected from a coal combustion plant in Manjung, Perak, Malaysia. The chemical composition of fly ash as analyzed using X-ray fluorescence (XRF) is tabulated in Table 1. The fly ash has total SiO_2_ and Al_2_O_3_ composition of 53.5%. A mixture of sodium hydroxide (NaOH) solution and liquid sodium silicate (Na_2_SiO_3_) was used as the alkaline silicate solution. The NaOH flakes were purchased from Formosa Plastic Corporation, Taiwan with a purity of 99%. The liquid sodium silicate was purchased from the South Pacific Chemical Industries Sdn. Bhd., Malaysia with chemical composition of 30.1% SiO_2_, 9.4% Na_2_O and 60.5% H_2_O (SiO_2_/Na_2_O ratio of 3.20). The foaming agent used was hydrogen peroxide (H_2_O_2_) with 30% w/w, purchased from R&M chemicals, United Kingdom.

### Preparation of Unfoamed and Foamed geopolymers

The NaOH 12 M solution was prepared and allowed to cool down to room temperature. The alkali activator was prepared one day before use by mixing 58.92 g of liquid Na_2_SiO_3_ and 23.52 g of NaOH solution. To synthesize unfoamed geopolymers, 165 g of fly ash was mixed with the alkali activator in a mixer until a homogeneous paste was obtained. Then, the geopolymer paste was compacted in 50-mm moulds and cured at room temperature until the day of testing. In order to synthesize porous geopolymer foam, the hydrogen peroxide (0.75 wt.%) was added and stirred followed by moulding and curing process at room temperature.

### Elevated Temperature Exposure

The 28 days-cured geopolymers were heated in a furnace at 200 °C, 400 °C, 600 °C, and 800 °C with a heating rate of 10 °C/min and soaking time of 2 hours. For comparison, one set of the samples was kept at ambient temperature (29 °C).

### Testing and Analysis Methods

The unfoamed and foamed geopolymers were tested with their compressive strength using Instron machine series 5569 Mechanical Tester as accordance to ASTM C109/109M-05. The aim of this test is to evaluate the performance of geopolymer when exposed to the elevated temperatures. Three samples were tested for each parameter. The bulk density of samples was measured prior and after exposure to elevated temperature by measuring the dimension and mass of the samples. The microstructural changes of fly ash, unexposed and exposed fly ash geopolymers were revealed using JSM-6460LA model Scanning Electron Microscope (JEOL) utilizing secondary electron detectors. For microstructural analysis of fly ash geopolymers, the specimen was the cut section prior to compressive strength. The thermal behaviour of geopolymers was analyzed with Perkin Elmer, Pyris diamond thermogravimetric analyzer (TGA) between 25 °C and 900 °C at a heating rate of 5 °C/min. Shifting of functional groups from fly ash toward fly ash geopolymers were identified using spectrum RXI spectrometer. The specimen was powdered samples scanned from 4000–650 cm^−1^ at the resolution of 4 cm^−1^. The phase analysis was conducted by using XRD-6000, Shimadzu X-ray diffractometer. Specimen for analysis was prepared in powder form. The XRD analysis was performed using Cu Kα radiation scanning from 2θ values in the range of 10° to 80° at a scan rate of 2° per minutes and scan steps of 0.02° (2θ). The XRD pattern was analyzed using X’pert HighScore Plus software equipped with ICDD PDF-2 database.

## Result and Discussion

### Properties of Unfoamed and Foamed Geopolymers

The unfoamed geopolymer achieved compressive strength of 44.2 MPa with bulk density of 2077 kg/m^3^ (Table 2). As expected, the bulk density of foamed geopolymers reduced with the addition of hydrogen peroxide. Oxygen gas released during the decomposition of H_2_O_2_ resulted in the porous structure. Thus, the foamed geopolymer had a lower bulk density of 1470 kg/m^3^ with lower strength of 15 MPa (Table 2). The bulk density of foamed geopolymer depended on the foaming agent content. Lower bulk density in the range of 500–750 kg/m^3^ was reported by Palmero *et al*.^31^ with 1–2% of H_2_O_2_ content. In this study, a lower content of H_2_O_2_ (0.75 wt.%) was applied which logically accounted for the higher bulk density.

The strength result of foamed geopolymers decreased 34% compared to that of unfoamed geopolymer. The compressive strength can be related to the decreased bulk density^20^. However, the strength results recorded in this study was much higher compared to those obtained by Masi *et al*.^5^ for geopolymers foamed with H_2_O_2_ (2.9–4.7 MPa) with comparable bulk density in the range 1120–1400 kg/m^3^. In addition, Sanjayan *et al*.^36^ reported that aerated geopolymer paste with Al powder exhibits low strength in the range of 0.9–4.35 MPa and the strength. Strength within 2.9–9.3 MPa has also been reported for H_2_O_2_-foamed fly ash geopolymers^6^.

### Elevated Temperature Exposure

Exposure at elevated temperature caused changes in bulk density and hence the mechanical strength of both unfoamed and foamed geopolymers. In general, all geopolymers exposed to heating retained cubic shape up to 800 °C without showing any destruction or dimensional change. This was supported by Skvara *et al*.^29^ for fly ash geopolymers. However, according to Badanoiu *et al*.^34^, the foamed geopolymers from glass and red mud experienced partial melting and softening.

**Figure 1:**
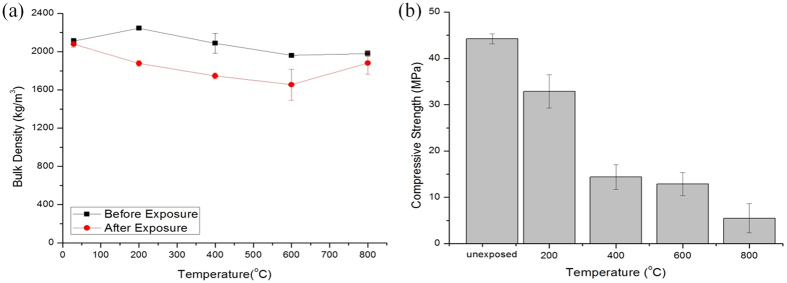
(**a**) Bulk density measurement and (**b**) compressive strength of unfoamed fly ash geopolymers before and after exposure to elevated temperature.

**Figure 2:**
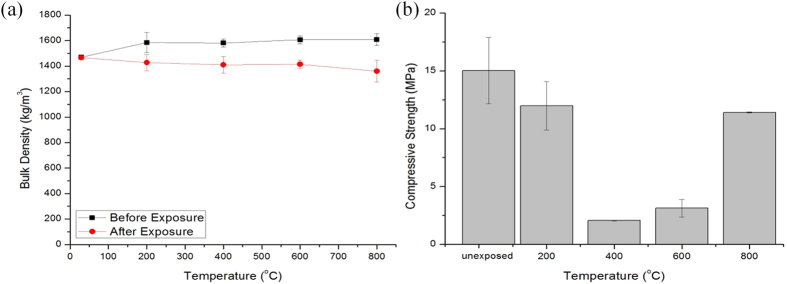
(**a**) Bulk density and (**b**) compressive strength of unexposed and exposed foamed fly ash geopolymers.

The unexposed geopolymers kept at room temperature showed little decrease in bulk density (Figs 1a and 2a). This was attributed the little moisture loss by evaporation during the curing process under room temperature. For unfoamed geopolymers, the decrease in bulk density was higher for samples heated at 200 °C, 400 °C and 600 °C which was about 16% mass loss (Fig. 1a and Table 3). This was due to the thermal shrinkage of geopolymer samples at elevated temperature^29^ as result of the liberation of water from the structure^36^,^37^ which weakened the geopolymer structure. Comparatively, the mass loss of geopolymers at 800 °C was lower (5%). This might because of the swelling of samples as result of heat or densification of geopolymer matrix that compensates the mass loss. This trend was supported by Duxson *et al*.^38^ whereby the geopolymer sample shrunk at the beginning of temperature exposure and finally densified at a higher temperature.

On the other hand, for foamed geopolymer, the mass losses were 9.9%, 10.8%, 12.0% and 15.4% at 200 °C, 400 °C, 600 °C and 800 °C, respectively, as refer to samples before exposure (Fig. 2a and Table 3). Higher exposing temperature resulted in a higher mass loss. However, the reduction in bulk density was not very significant. The variation of weight change of foamed geopolymers was smaller compared to cement foam^29^. The mass loss for both unfoamed and foamed geopolymers was relatively similar. However, the mass loss for foamed geopolymer was slightly lower. The difference in the water content of geopolymers contributed to the dissimilarity of density reduction. Supported by the TGA curves in the section below, foamed fly ash geopolymers had less water content. Even so, the mass loss of geopolymer after exposure to temperature was considered small (9.9–16.4%). According to Luna-Galiano *et al*.^39^, OPC samples had a higher mass loss (20%) than geopolymers and therefore greater degradation with rising temperature. On due course, geopolymer was deemed to have better structural integrity than OPC products.

**Figure 3:**
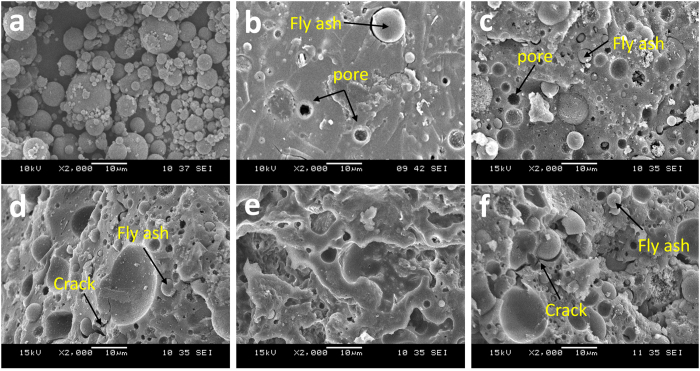
SEM micrographs of (**a**) fly ash; (**b**) unexposed and exposed unfoamed fly ash geopolymers at (**c**) 200 °C; (**d**) 400 °C; (**e**) 600 °C; and (**f**) 800 °C.

The strength of unfoamed geopolymers reduced from 32.9 MPa to 5.5 MPa with strength loss from 25.7% to 87.6% upon heating from 200 °C to 800 °C (Fig. 1b and Table 3). This complied with the decreased bulk density of geopolymers with rising temperatures. The strength decreased at a slower rate after 400 °C. As stated above, the loss of water with increasing temperature led to more pores (Fig. 3) in structure and consequently lower strength^40^. The reduction of geopolymer strength at elevated temperature was also concurred by Zhang *et al*.^26^ and Zuda *et al*.^41^ up to 800 °C. In addition, Lemougna *et al*.^21^ also observed the similar downward strength trend for dense volcanic ash geopolymers thermally-treated in the temperature range of 250 °C–900 °C. A contrast result was reported by Bakharev^42^ wherein the firing of fly ash geopolymer tended to reduce the average pore size and improve the compressive strength. Generally, fly ash geopolymers exhibited higher strength degradation^26^. The addition of metakaolin in fly ash geopolymer could increase the thermal performance. However, the strength retention of purely fly ash geopolymers was still higher than that of purely metakaolin geopolymers^22^. Referring to Fig. 1b, at 800 °C, the strength did not increase even it had the lowest mass loss. This can be explained by the distortion of structure and geopolymer matrix^25^. The migration of water to the surface at elevated temperature induced internal damage to the overall structure of the geopolymer. At the same time, expansion in the geopolymers samples also degraded the strength of geopolymers.

As refer to Fig. 2b and Table 3, the compressive strength of foamed geopolymers degraded 20.2% (12 MPa) when exposed at 200 °C compared to unexposed geopolymer foams. The strength reduced significantly with 86.3% (2 MPa) and 79.2% (3 MPa) strength loss when heated at 400 °C and 600 °C, respectively. In another way, samples heated at 800 °C retained higher strength of 11 MPa (24% strength loss). Dissimilar strength trend was obtained by Zhang *et al*.^20^ as the foamed fly ash geopolymers retained its strength when heated to 400 °C and further increase in strength higher than those of unexposed geopolymer foams at 800 °C. It was believed that the relatively higher SiO_2_ and Al_2_O_3_ contents in the fly ash used in the study accounts for the formation of more Si-Al matrix. The Si-Al rich matrix had better strength retention^43^. Although the geopolymers reduced in strength after the thermal treatment, the strength of >2 MPa was adequate for foamed materials^34^. On the other hand, based on Bernal *et al*.^43^, foamed metakaolin geopolymer reduced in strength up to 800 °C and further increased after 1000 °C. For slag-metakaolin foamed geopolymers, they tended to reduce in strength consistently from 200 °C to 1000 °C^18^. It is pronounced that the strength deterioration of foamed geopolymer was generally lower than unfoamed geopolymer. The pore structure of foam material was expected to allow the heat transfer and minimizing the thermo-mechanical damage^28^. The porosity allowed fast removal of water leading to enhanced thermal resistance^25^,^30^. This statement was further supported by Zhao & Sanjayan^44^ who stated that internal pore structure allowed quick escape of water vapour that reduced the pore pressure.

According to previous literature^26^, the mass loss of geopolymers and thermal deformation due to water evaporation determined the performance of geopolymers with increasing temperature. However, the data of this experiment was unlikely following the similar trend. Despite this, the internal damage resulted from the evaporating water greatly affected the strength retention of geopolymers. When temperature increased, water inside the pore cavities or the structural water rapidly migrated and evaporated through the surface. This movement of moisture induced internal damage to the microstructure of specimen and consequently deteriorated the strength. As refer to Table 3, the unfoamed and foamed geopolymers revealed different variation of mass and residual strength. Although similar to OPC concrete, geopolymers experienced strength deterioration when subjected to elevated temperature; the strength retention of geopolymer was much greater than that of OPC. The OPC paste was totally damage at elevated temperature^23^,^26^,^45^ due to evaporation of water and decomposition of hydrate products^1^,^39^.

### Microstructural Analysis

Figure 3a Shows class F fly ash particles. It reveals that the morphology of fly ash particles as spherical-shaped particles with smooth surfaces. Figure 3b–f reveals the microstructure of unexposed and temperature-exposed unfoamed geopolymers. Unexposed fly ash geopolymer matrix (Fig. 3b) appeared smooth with some remnant fly ash particles. Few coarse pores can be seen in the geopolymer matrix. After the exposure to elevated temperature, the increase in the porosity distributed throughout the matrix can be clearly seen in the matrix. This confirmed the decreased bulk density as shown in Fig. 1a. The observation was also attested by Badanoiu *et al*.^34^. With increasing temperature, the pores became slightly larger. These pores might have left behind by the escaping water during the temperature exposure or dissolution of remnant fly ash particles. Besides, the porosity increment might probably because of the thermal shrinkage resulted from thermal damage in geopolymers^39^.

Smooth geopolymer matrix can still be seen in samples heated at 200 °C (Fig. 3c) and 400 °C (Fig. 3d) with the presence of small cracks. In samples treated at 600 °C, sintering effect was shown by the formation of connecting matrix (Fig. 3e). On the other hand, for samples exposed to 800 °C, the appearance of intervening matrix was not seen but greater deterioration by heat was observed (Fig. 3f) with larger cracks and loose microstructure. This was believed the main factor causing the significant decrease in strength of unfoamed geopolymers. The observation on the microstructure of fly ash geopolymer was supported by Omar *et al*.^36^. Unfortunately, the fly ash geopolymer paste experienced a total loss of strength.

**Figure 4:**
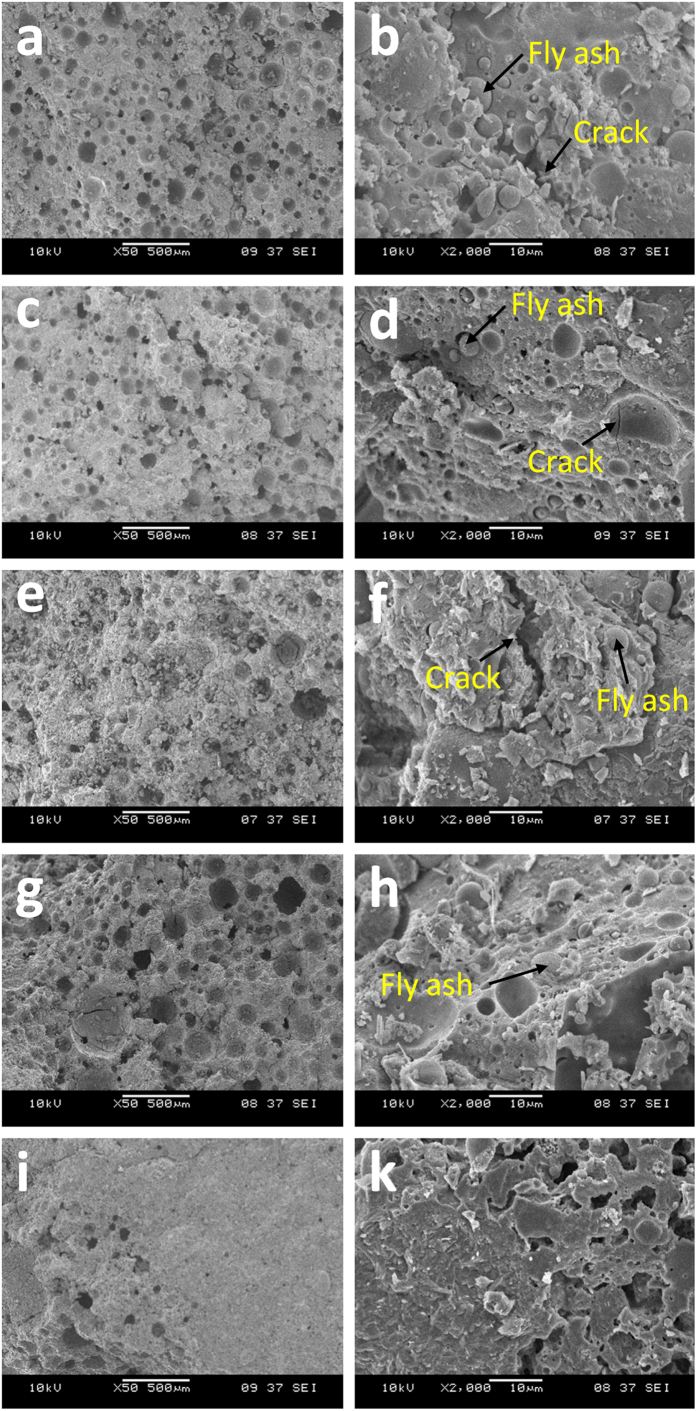
SEM micrograph of unexposed (**a**,**b**) and temperature-exposed foamed fly ash geopolymers at 200 °C (**c**,**d**), 400 °C (**e**,**f**), 600 °C (**g**,**h**) and 800 °C (**i**,**j**).

Figure 4 Reveals the SEM micrographs of unexposed and exposed foamed geopolymer to elevated temperature at different magnification. SEM micrographs of foamed geopolymers were taken at magnification of 50× and 2000×. Foamed fly ash geopolymers were obtained as the effect of the addition of hydrogen peroxide. Pores were evenly distributed within the matrix^31^. According to Masi *et al*.^5^, a more homogeneous distributed macro-pores can be obtained if the sample was foamed with surfactant rather than using H_2_O_2_.

At lower magnification, small cracks can be observed in all samples exposed to elevated temperature. Relatively smooth geopolymer matrix was revealed in unexposed foamed geopolymer (Fig. 4b). Comparatively, the micrographs of the sample heated at 400 °C (Fig. 4c) and 600 °C (Fig. 4e) appeared loose and unconnected. Large cracks can be seen in 400°C-exposed samples (Fig. 4f). This was most probably responsible for the lowest strength obtained (Fig. 2b). In the contrary, sample heated at 800 °C revealed lesser pores (Fig. 4i) even though the mass loss was the highest. The geopolymer matrix intervened with each other forming continuous matrix at large magnification (Fig. 4j). This suggested that there is sintering effect and partial melting due to elevated temperature. The partial melting which allowed the viscous flow to fill pores or voids present in the structure^27^. The sintering effect caused smaller strength drop in 800 °C compared to other samples. However, it did not lead to enhanced strength as previously reported by Skvara *et al*.^29^. The matrix was seemingly dense with crystalline phases, which is estimated to improve the strength of foamed geopolymer at 800 °C. The presence of crystalline phases was confirmed by the XRD diffractogram in the section below. Thus, lower strength loss was observed for this sample. Even so, non-connected small pores can be observed in the microstructure.

At the same time, some remnant fly ash particles were left as indicated by the spherical-shaped particles. With increasing temperature exposure, the amount of residual fly ash particles became lesser and disappeared in samples heated at 800 °C.

### Thermogravimetric Analysis

**Figure 5:**
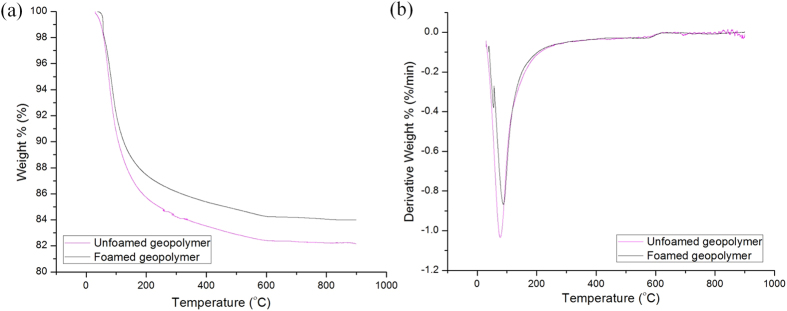
(**a**) TGA and (**b**) DTG curves of fly ash geopolymers.

Figure 5 Illustrates the TGA and DTG curves of unfoamed and foamed fly ash geopolymers. The curves showed mass loss of geopolymers in presence of heat. Sharp reduction of mass occurred below 200 °C (approximately 70% of the total water content). It was mainly associated with the evaporation of evaporable “free” water weakly adsorbed in the structure and cavities of the structure^29^. This value was commonly reported^21^. The mass loss rate slowed down after 200 °C whereby the little decrease in mass loss occurred after 200 °C was due to the chemically bonded water and OH groups^15^. The decomposition of sodium carbonate started at 400 °C^21^. No mass change was observed after 600 °C up to 800 °C, which was also observed by Zhang *et al*.^26^ in metakaolin-fly ash based geopolymers.

The unfoamed and foamed geopolymers contained 18% and 16% of water content, respectively. The masses remaining when heated up to 800 °C were 82% for unfoamed geopolymer and 84% for foamed geopolymers. The water loss complied with the mass loss result (Table 3). The foamed geopolymers experienced lesser loss of water compared to unfoamed geopolymers. The overall lesser water content in samples accounted for the better structural stability of foamed geopolymers than unfoamed geopolymers. Comparatively, the mass loss of fly ash geopolymers was lower than that of metakaolin geopolymers when exposed to elevated temperatures. This was because metakaolin geopolymers usually had higher liquid/solid ratios^26^,^46^. As compared to OPC, Fernandez-Jimenez *et al*.^19^ observed three peaks at around 97°, 450° and 750° where the first peak was attributed to the loss of water and decomposition of C-S-H gel and the latter two peaks were associated with the portlandite and CaCO_3_ decomposition.

### FTIR Analysis

**Figure 6:**
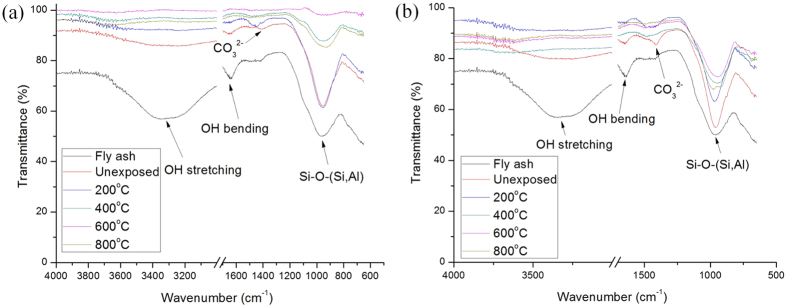
FTIR spectra of unexposed and exposed (**a**) unfoamed and (**b**) foamed fly ash geopolymers to elevated temperature.

Figure 6 Represents the FTIR spectra of unfoamed and foamed geopolymers exposed to elevated temperature. Fly ash showed main broad absorption band at ~960 cm^−1^, which corresponding to the asymmetrical stretching vibration of Si-O-Si and Si-O-Al. This band shifted to lower wavenumber (~950 cm^−1^) after the geopolymerization reaction^33^. This inferred the incorporation of Al atoms in the silicate geopolymer network and increasing non-bridging oxygen in structure^32^,^47^. The shifting of the band also indicated formation of larger molecular structure and higher cross-linking^21^,^48^. The other absorption bands at ~3300 cm^−1^ and ~1640 cm^−1^ were attributed to the OH stretching vibration and bending vibration, respectively. The band at ~1470 cm^−1^ was the CO_3_^2−^ ion resulted from the reaction of atmospheric CO_2_ with residual sodium content^49^. With increasing temperature, the sodium carbonate band disappeared as it started to decompose at 400 °C, as aforementioned. The FTIR absorption bands were summarized in Table 4. When heated at elevated temperature, no additional phase changes or formation of new bands were observed. Similar to many other geopolymers exposed to heat treatment^50^, the bands at ~3300 cm^−1^ and ~1640 cm^−1^ lowered in intensity with increasing temperature exposure, indicating fully dehydration of geopolymers.

### XRD Analysis

**Figure 7:**
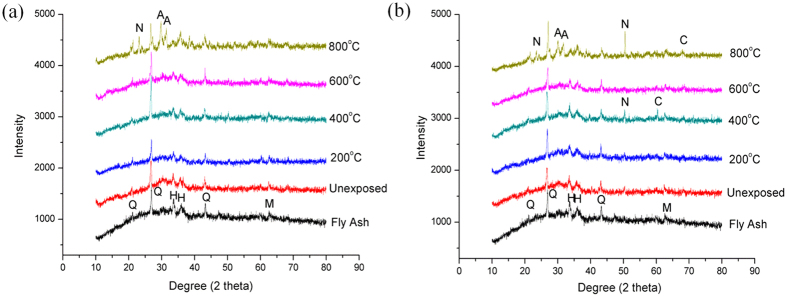
XRD diffractograms of fly ash, unexposed and exposed unfoamed and foamed fly ash geopolymers (Q – Quartz (PDF no. 01-078-1255), H – Hematite (PDF no. 01-073-0603), M – Magnetite (PDF no. 01-079-0418), N – Nepheline (PDF no. 00-009-0338), A – Anorthite (PDF no. 00-020-0020) and C – Cristobalite (PDF no. 00-004-0379)).

Figure 7 Presents the XRD diffractograms of fly ash, unfoamed and foamed geopolymers. Fly ash showed broad humps of amorphous phase at 2θ between 15–38° with some crystalline phases of quartz (SiO_2_), hematite (Fe_3_O_4_) and magnetite (Fe_3_O_4_). After the reaction with alkali silicate solution, the diffuse hump of fly ash shifted slightly towards higher degree (20°–40° 2θ). This was the typical characteristic of aluminosilicates matrix and the shift indicated the formation of geopolymer matrix after the geopolymerization reaction^32^. Mineral phases of quartz, hematite and magnetite were still present in geopolymers but with slightly reduced intensity. This reflected that not all mineral phases are participating in the geopolymerization reaction towards the formation of geopolymer matrix or the limited dissolution of the mineral phases.

The elevated temperature increased the propensity towards the formation of stable crystalline phases. Formation of nepheline, anorthite and cristobalite could be seen in geopolymers at higher temperature exposure. The crystalline phases were mostly found in samples heated at 800 °C. In general, the crystallization would most probably enhance the mechanical properties of geopolymers^51^. Crystalline phases might act as fillers to reinforce the geopolymer matrix. However, from the result of this study, the formation of crystalline phases was supposed inducing thermal stress within the geopolymers and deteriorated the strength in the case of unfoamed geopolymers^20^. The thermal stress was believed caused by the thermal mismatch between matrix and the crystalline phases. The geopolymer matrix shrunk while the crystalline phases expanded upon heat-treated and thus formed large cracks as seen in Fig. 3f. In the other way, the formation of crystalline phases was believed inducing less damage to foamed geopolymers as the porosity provides ‘rooms’ for the expansion, causes less disruption to the final structure and finally reinforces the structures^27^. The presence of the crystalline phases that remained stable at high temperatures is important for the thermal stability.

## Conclusion

This paper presented the characteristic of unfoamed and foamed fly ash geopolymer exposed to elevated temperatures. No disintegration and spalling of geopolymer samples occurred when subjected to high temperature. Unexposed unfoamed geopolymer exhibited excellent compressive strength (44.2 MPa) with density ranged between 1900 and 2200 kg/m^3^. With the addition of hydrogen peroxide as foaming agent, foamed geopolymer was obtained. However, the strength reduced 34% from that of unfoamed geopolymer with lower bulk density (1400–1600 kg/m^3^).

When the geopolymers are heated at elevated temperature, the bulk density and compressive strength decreased for unfoamed geopolymers. On the other hand, the compressive strength of geopolymer foam decreased when heated to 400 °C and in other way rises up to 800 °C. It was deemed that the fast evaporation of water from the geopolymer structure induces internal damage to the structure within. Based on SEM analysis, pores in unfoamed geopolymer increased due to loss of water. More intervening geopolymer matrix could be observed at higher temperature exposure due to sintering and partial melting. Besides, according to the XRD analysis, nepheline, cristobalite and anorthite crystalline phases formed at high temperature exposure were deleterious to unfoamed geopolymers but beneficial to geopolymer foams. From this study, it could be concluded that unfoamed fly ash geopolymer had lower resistance towards elevated temperature. Foaming of geopolymers helped to minimize the disruption effect caused by thermal treatment as the pores provides room to counteract the damage by heat.

## Additional Information

**How to cite this article:** Cheng-Yong, H. *et al*. Thermal Resistance Variations of Fly Ash Geopolymers: Foaming Responses. *Sci. Rep.* **7**, 45355; doi: 10.1038/srep45355 (2017).

**Publisher's note:** Springer Nature remains neutral with regard to jurisdictional claims in published maps and institutional affiliations.

## Figures and Tables

**Table 1:** Chemical composition of class F fly ash.

Compound	SiO_2_	Al_2_O_3_	Fe_2_O_3_	TiO_2_	CaO	MgO	SO_3_	K_2_O	MnO	BaO	SrO
Mass (wt.%)	38.80	14.70	19.48	1.02	18.10	3.30	1.50	1.79	0.16	0.27	0.11

**Table 2:** Compressive strength and bulk density of unfoamed and foamed geopolymers.

	Strength (MPa)	Bulk density (kg/m^3^)
Unfoamed geopolymer	44.2 ± 1.1	2077 ± 30.9
Foamed geopolymer	15.0 ± 2.9	1470 ± 15.5

**Table 3:** Mass and strength losses of unfoamed and foamed geopolymers.

Temperature (°C)	Unfoamed Geopolymers		Foamed Geopolymers	
	Mass Loss (%)	Strength Loss (%)	Mass Loss (%)	Strength Loss (%)
29	1.6	—	0.7	—
200	16.4	25.7	9.9	20.2
400	16.4	67.4	10.8	86.3
600	15.7	70.9	12.0	79.2
800	5.1	87.6	15.4	24.0

**Table 4:** Assignment of main FTIR bands.

Band	Assignments
3300 cm^−1^	OH stretching vibration
1640 cm^−1^	Bending vibration of H-O-H
1470 cm^−1^	Stretching vibration of CO_3_^2−^ ion
960 cm^−1^	Asymmetrical stretching vibration of Si-O-Si and Si-O-Al
940 cm^−1^	Asymmetrical stretching vibration of Si-O-Si and Si-O-Al
